# 170. Intrapartum Antibiotic Exposure and Infectious Diseases in Childhood - A Population-based Cohort Study

**DOI:** 10.1093/ofid/ofae631.050

**Published:** 2025-01-29

**Authors:** Mikael Hakkola, Sofia Ainonen, Eveliina Ronkainen, Minna Honkila, Marika Paalanne, Tytti Pokka, Eero Kajantie, Niko Paalanne, Terhi Ruuska

**Affiliations:** University of Oulu, Oulu, Pohjois-Pohjanmaa, Finland; University of Oulu, Oulu, Pohjois-Pohjanmaa, Finland; University of Oulu, Oulu, Pohjois-Pohjanmaa, Finland; University of Oulu, Oulu, Pohjois-Pohjanmaa, Finland; University of Oulu, Oulu, Pohjois-Pohjanmaa, Finland; University of Oulu, Oulu, Pohjois-Pohjanmaa, Finland; University of Oulu, Oulu, Pohjois-Pohjanmaa, Finland; University of Oulu, Oulu, Pohjois-Pohjanmaa, Finland; University of Oulu, Oulu, Pohjois-Pohjanmaa, Finland

## Abstract

**Background:**

Universal maternal screening for group B streptococcus (GBS) colonization and subsequent use of intrapartum antibiotics is currently recommended practice in many high-income countries. This strategy has reduced the occurrence of early-onset GBS diseases in infants significantly. However, this practice exposes up to 30% of infants to antibiotics at birth. Intrapartum antibiotics have been shown to alter the gut microbiome composition in the offspring, which may be associated with long-term clinical outcomes. We hypothesized that maternal use of intrapartum antibiotics increases the risk of infectious diseases in the offspring.

**Table 1.**
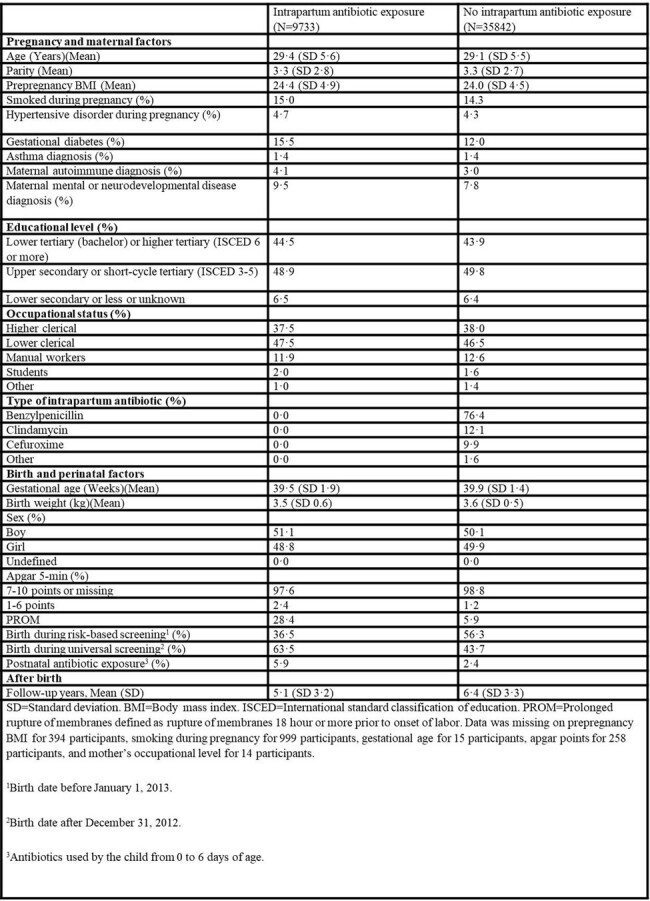
Baseline characteristics of the study cohort

**Methods:**

In a cohort study of 45 575 vaginally delivered newborn infants in Finland, there were 9733 children exposed to intrapartum antibiotics and 35 842 unexposed children. Intrapartum antibiotic exposure was defined as antibiotics administered to mother 24 hours or less before birth of the child. The data used in the study were retrieved from high quality national registers and electronic medical records. Primary outcome was the annual number of infectious disease episodes leading to hospitalization, emergency department visit, or other outpatient visit in specialized health care during the first five years of life.

**Table 2.**
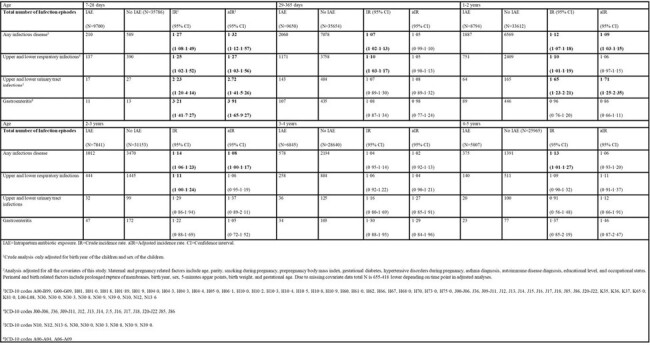
Number of infectious disease episodes during the first five years of life.

**Results:**

Intrapartum antibiotic exposure was associated with increased risk of any infectious disease episode at ages 7-28 days (adjusted incidence rate [aIR)] 1.32, 95% CI 1.12−1.57), 1−2 years (aIR 1.09, 95% CI 1.03−1.15), and 2−3 years (aIR 1.08, 95% CI 1.00−1.17). Intrapartum antibiotic exposure was also associated with an increased cumulative incidence of at least one infectious disease episode, increased number of urinary tract infection episodes, and increased number of antibiotic courses received. We found no association with severe infections. Intrapartum antibiotics protected offspring from severe infections caused by pathogens susceptible to penicillin beyond neonatal period.

**Table 3.**
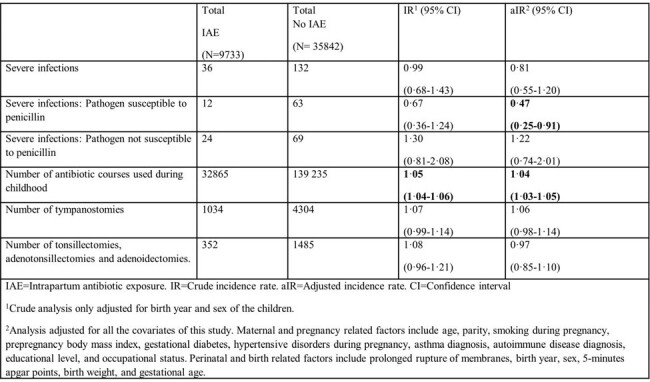
Number of severe infection episodes, number of antibiotics courses used and number of infection related surgical operations performed during the first five years of life.,

**Conclusion:**

Intrapartum antibiotics were associated with a long-term altered risk of infectious diseases in childhood. The observed associations appeared to depend on the causative pathogens and their susceptibility to penicillin. Previously observed changes in gut microbiome might explain the findings.

**Figure 1.**
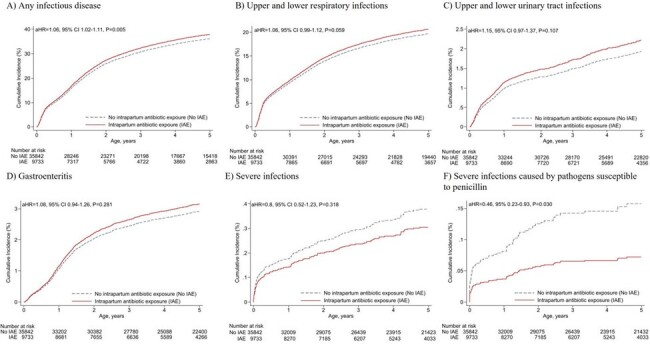
Cumulative incidence of at least one infectious disease episode during the first five years of life.

**Disclosures:**

**All Authors**: No reported disclosures

